# Supplementary material for: Qualitative Evaluation of Causes for Routine Salmonella Monitoring False-Positive Test Results in Dutch Poultry Breeding Flocks
Source: Microorganisms. 2021 Oct 25;9(11):2215. doi: 10.3390/microorganisms9112215 (PMC8620518; doi:10.3390/microorganisms9112215)
Supplement: Supplementary file 1 [file microorganisms-09-02215-s001.zip › microorganisms-1398058-supplementary.pdf]

Supplementary material A

Table S1. Questionnaire on the potential for contamination of samples during the process of *Salmonella* testing in poultry breeding farms.

Name:<br>Country:										
Q1: In your experience as a poultry practitioner, do you think that it is possible to get a false positive *Salmonella* result from a farm because samples taken by the farmer have become contaminated through cross-contamination during sampling?									( ) Yes ( ) No	
How sure are you about your statement regarding Q1 (please indicate on a scale from 1-10)?										
0<br>Not<br>sure	1	2	3	4	5	6	7	8	9	10<br>Very<br>sure
Q2: In your experience as a poultry practitioner, do you think that it is possible to get a false positive *Salmonella* result from a farm because samples taken by the farmer have become contaminated through cross-contamination during transport of the samples from the farm to the laboratory?									( ) Yes ( ) No	
How sure are you about your statement regarding Q2 (please indicate on a scale from 1-10)?										
0<br>Not<br>sure	1	2	3	4	5	6	7	8	9	10<br>Very<br>sure
Q3: In your experience as a poultry practitioner, do you think that it is possible to get a false positive *Salmonella* result from a farm because samples have become contaminated through cross-contamination in the process of handling and isolation in the lab?									( ) Yes ( ) No	
How sure are you about your statement regarding Q3 (please indicate on a scale from 1-10)?										
0<br>Not<br>sure	1	2	3	4	5	6	7	8	9	10<br>Very<br>sure
Q4: Do you think that, given the existence of false positive *Salmonella* results in practice, it is important that all initial positive *Salmonella* results from a farm are confirmed by resampling and retesting from the same barn and farm?									( ) Yes ( ) No	
How sure are you about your statement regarding Q4 (please indicate on a scale from 1-10)?										
0<br>Not<br>sure	1	2	3	4	5	6	7	8	9	10<br>Very<br>sure

If you have answered questions Q1 to Q3 with "Yes", what would be your ranking of the likelihood of the appearance of false positives?			
	Q1 (Sampling)	Q2 (Transport)	Q3 (Laboratory)
Ranking 1 (most important)			
Ranking 2 (moderately important)			
Ranking 3 (less important)			

Supplementary material B

Protocol S1. Description of sampling protocol by the poultry farmer.

Based on a translation from a flyer on AVINED website: https://www.avined.nl/thema/monstername-salmonella-leghennen.

Specific description for boot swab samples.

1. With washed/clean hands, moisten the surface of both specimens of a pair of boot swabs. The boot swabs should be made of absorbent material.

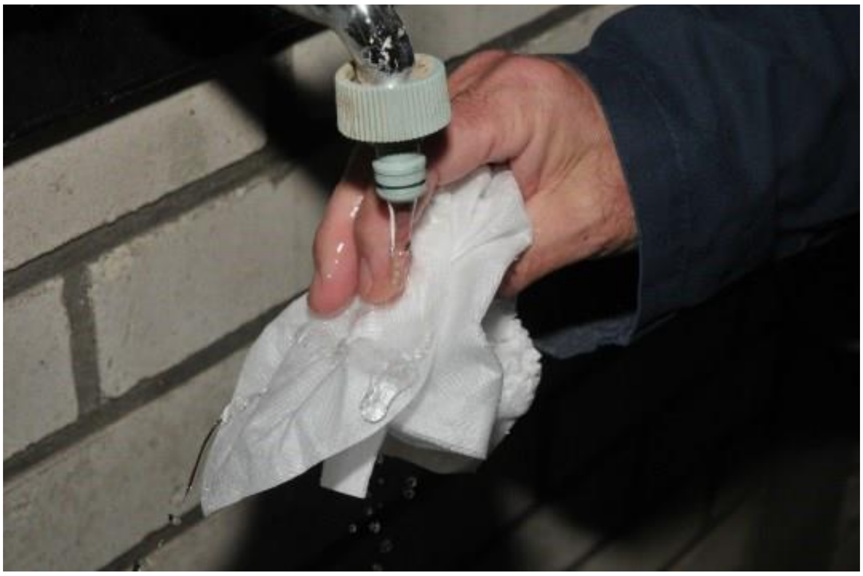

2. For each poultry house, put a pair of boot swabs over your (rubber) boots and walk around through the entire stable (to be repeated with several pairs of boot swabs).

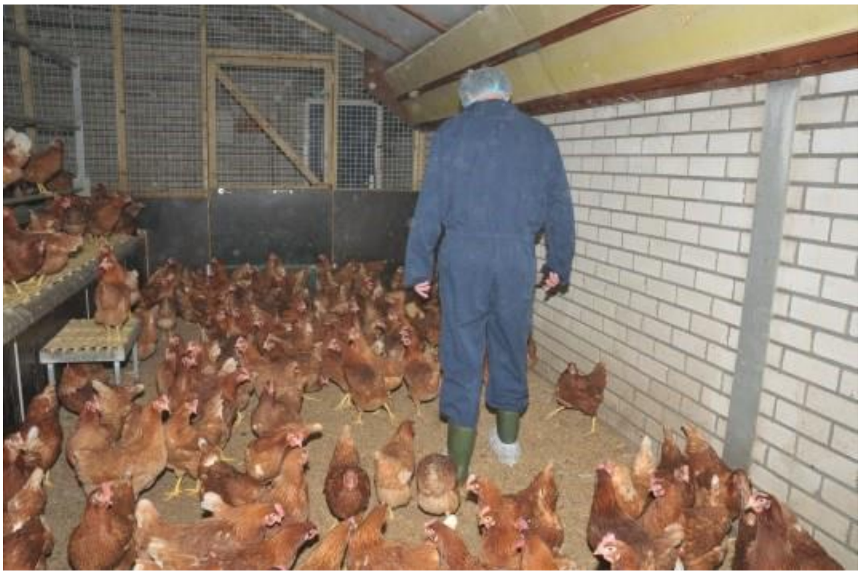

3. When leaving the poultry house, put the boot swabs used for sampling in a sterile plastic bag. The bag must be provided with the name and address details of the holder, sampler, farm number, date of sampling, poultry house number, date of birth of flock, farm type, sample type and name and signature of the sender.

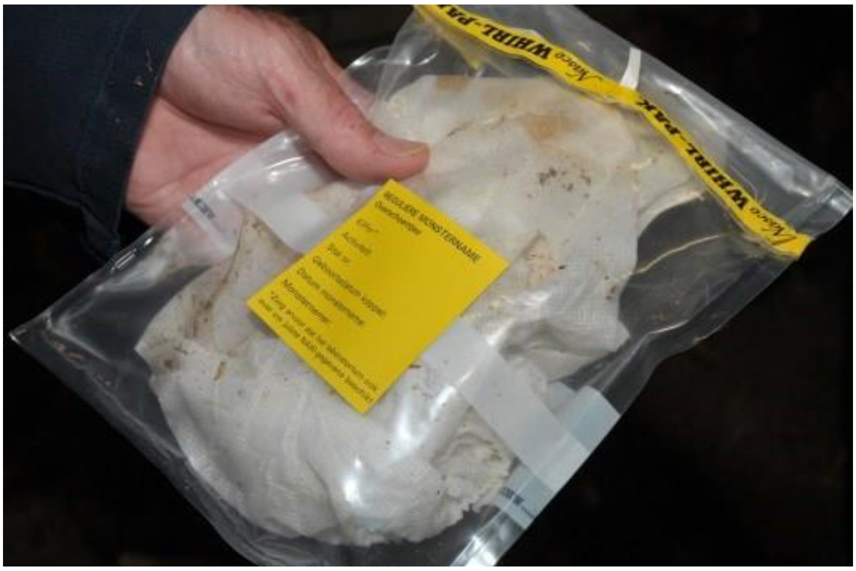

4. Samples must be sent as soon as possible (next day at the latest) to an accredited laboratory.

Supplementary material C

Table S2. Work instruction handling suspicion of zoonotic *Salmonella* at the poultry farm.

Based on a translation of the work instruction VERD PRO 1216v of the Netherlands Food and Consumer Product Safety Authority (NVWA). The instructions for official samples taken by veterinarians to confirm a

case of a positive flock in the Netherlands are divided into three steps: i) preparing the visit, ii) visit at the location, and iii) finishing the visit.

Preparing the visit

Preparation actions	Considerations
Receive the notification	Receive the notification and case order from NVIC by phone or email.
Print out the information	Print out the work order and the attachments, bringing it all with you.
Inform the farmer	Inform the farmer about the suspected case of *Salmonella* infection and to declare eggs and animals to be officially in isolation until the results of the investigation are known
Make an appointment with the farmer	Schedule a date to collect samples from the suspected poultry houses. Ask for the identification of the poultry houses.
Prepare the sampling and inform NVIC	Inform the NVIC about the time and location of the sampling.
Order/require the sample materials	The containers with boot swabs, including peptone water are delivered to your home via the NVIC. Make sure you have enough large disposable plastic overshoes, disposable plastic gloves, etc.
Check required PBM's	Check the hygiene instructions sheet and take it with you.
Sample form	Fill an individual sample form per poultry house. Use the RAA number for each barn.

Visit at location

Preparation actions	Considerations
Arrival at the farm, consider hygiene and PPE (private protection equipment) use	Consult the work instructions for hygiene and occupational health and safety. Also, follow the biosecurity measures from the farm.
Counting and classification	Check the number of animals in the poultry house with the data from the farm-system. Register it on the visit report.
Check the records of antimicrobial use	Check in the logbook of the farm if antibiotics were used in animals of

	the suspected flock. If antibiotics were administered, check the information in prescription whether the waiting period has been expired or not. Besides that, 5 individual chickens have to investigated for antibiotic residues. When no antibiotics were administered, inform the farmer that the flock will be cleared from suspicion when the test results of the boot swabs are negative. The antibiotic residue investigation takes about 8-12 days. If antibiotic residues are detected during the investigation, the flock will be declared infected yet.
Fill in the visit report and the questionnaire	Note the unique identification number used on the eggs from the suspected poultry house. If eggs are not labeled with a unique identification number per poultry house, this must be stated on the visit report.
Call the currier	Warn/call the courier to be prepared to pick up the samples for transport to the lab
Sampling procedures<br>RAA0144037-01	Boot swabs (5 pairs per suspect poultry house)<br>• Put on coveralls provided by the farm. If this is not present, put on a second disposable coveralls.<br>• Put on plastic disposable overshoes over your socks before you put on the (rubber) boots provided by the farm.<br>• Before taking samples, wash your hands thoroughly with disinfectant soap and put on disposable gloves.<br>• 5 pairs of boot swabs are used per suspect poultry house.<br>• The boot swabs come in pairs in a jar with peptone water.<br>• Put a RAA sticker with a serial number on the outside of each jar. See image.<br>• Take a large seal bag or clean bucket containing the jars into the poultry house.

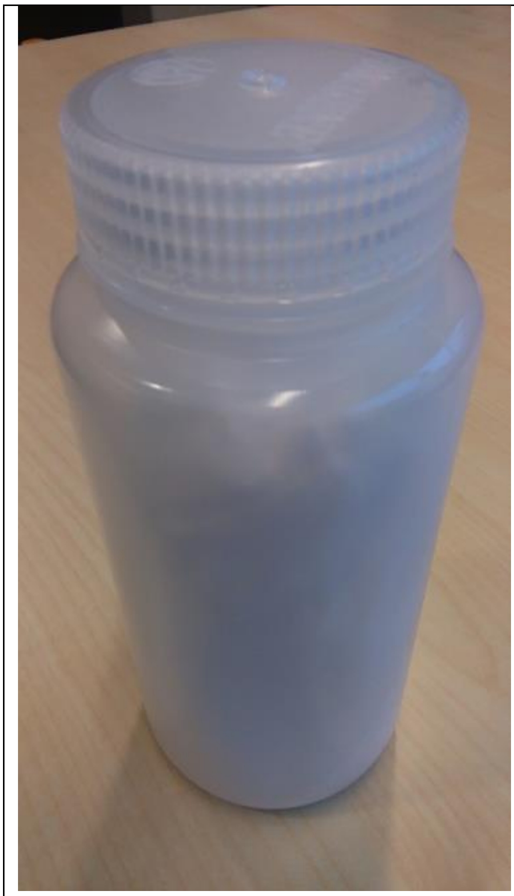	• After sample preparation put on a pair of clean disposable gloves.<br>• First, put on a pair of large plastic disposable overshoes in the stable over the farm boots.<br>• Put the 5 pairs of boot swabs over each other over the plastic disposable overshoes.<br>• Walk slowly through the poultry house over the parts with manure. Walk about a fifth of the area to be sampled, and pull off the outer pair of boot swabs. Fold the pair of boot swabs inside out, so that the adhering manure remains in the boot swab. Put the pair of boot swabs (1 pair) in a jar and close it immediately. Repeat this until the last pair of boot swabs are put in the last jar just before leaving the poultry house. You now have five jars with two boot swabs each.<br>• Put the 5 jars with the boot swabs in the supplied seal bag.<br>• Stick a label with the case number and poultry house number on the outside of the large seal bag. This poultry house number must match the poultry house numbers on the map.<br>If several poultry houses need to be sampled, wash your hands in between with disinfectant soap and use clean PPE.
Sampling procedure	**5 chickens**<br>In the suspect poultry house 5 chickens are selected and taken for testing for the presence of antibiotic residues.<br>The chickens are sent by courier to the reference lab, the courier will also take the boot swabs for transport.
Sample forms<br>RAA0144037-XX	Fill in a digital sampling form per poultry house. Use a new sheet of RAA stickers for each poultry house. State on the chicken sample form "Poultry scan".<br>Also, stick the barcode stickers with the XX code (for both the

	boot swabs and the chickens) on the "Report site visit".
Packaging of samples	**Boot swabs**<br>The different jars with boot swabs are put in a large seal bag and closed.<br><br>**Chickens**<br>The chickens are transported alive in poultry crates to the reference lab
Address of reference lab	Put the correct address of the reference laboratory on the shipment; contact the reference lab by phone and report the shipment of samples
Floor plan	During the visit, describe the place properly or use the empty floor plan provided by NVIC. Cleary indicate the poultry house identificaions and collects the farmer's signature for confirmation.
To fil in forms	Make sure that the following items are filled: floor plan, visit report, attendance list, questionnaire for zoonotic *Salmonella* and digital sampling form.

Finish the farm visit

Preparation actions	Considerations
Results	Diagnostic results are known 8 to 10 days after the samples arrived at the reference lab. This is the maximum lead time for S. Enteritidis and Typhimurium serovars. If other serovars are suspected, then the isolate must be submitted to Institute of Public Health and the Environment (RIVM) and this investigation may take from 10 to 13 days.
PPE use	For the order in which to get out of coveralls, boots etc., please check the work instruction " hygiene and ARBO" (ALG PRO 1001)

Outer package samples	Use “Sterilium” to disinfect the outside of the seal bag with samples when leaving the farm.
Transfer samples to a courier	Hand over the samples to the courier yourself and sign digitally.
Sign out at NVIC	Report to NVIC by phone that you are leaving the farm.
Return all forms to NVIC	Scan the forms and send them as soon as possible by email, ultimately the next working day; return all forms to NVWA.
